# Detailed view on slow sinusoidal, hemodynamic oscillations on the human brain cortex by Fourier transforming oxy/deoxy hyperspectral images

**DOI:** 10.1002/hbm.24194

**Published:** 2018-04-25

**Authors:** H.J. Noordmans, D. van Blooijs, J.C.W. Siero, J.J.M. Zwanenburg, J.H.G.M. Klaessens, N. F. Ramsey

**Affiliations:** ^1^ FB Medical Technology and Clinical Physics, University Medical Center Utrecht The Netherlands; ^2^ Department of Neurology and Neurosurgery Brain Center Rudolf Magnus, University Medical Center Utrecht The Netherlands; ^3^ Department of Radiology University Medical Center Utrecht The Netherlands; ^4^ Spinoza Centre for Neuroimaging Amsterdam The Netherlands; ^5^ Department of Physics and Medical Technology VU University Medical Center Amsterdam The Netherlands

**Keywords:** cerebral cortex, epilepsy, hemodynamics, hemoglobins, humans

## Abstract

Slow sinusoidal, hemodynamic oscillations (SSHOs) around 0.1 Hz are frequently seen in mammalian and human brains. In four patients undergoing epilepsy surgery, subtle but robust fluctuations in oxy‐ and deoxyhemoglobin were detected using hyperspectral imaging of the cortex. These SSHOs were stationary during the entire 4 to 10 min acquisition time. By Fourier filtering the oxy‐ and deoxyhemoglobin time signals with a small bandwidth, SSHOs became visible within localized regions of the brain, with distinctive frequencies and a continuous phase variation within that region. SSHOs of deoxyhemoglobin appeared to have an opposite phase and 11% smaller amplitude with respect to the oxyhemoglobin SSHOs. Although the origin of SSHOs remains unclear, we find indications that the observed SSHOs may embody a local propagating hemodynamic wave with velocities in line with capillary blood velocities, and conceivably related to vasomotion and maintenance of adequate tissue perfusion. Hyperspectral imaging of the human cortex during surgery allow in‐depth characterization of SSHOs, and may give further insight in the nature and potential (clinical) use of SSHOs.

## 1 INTRODUCTION

When performing EEG, NIRS, or (resting state) fMRI, one of the factors attributing to the measurements are slow sinusoidal hemodynamic oscillations (SSHOs) around 0.1 Hz.^1^ These SSHOs are hypothesized to have two possible origins: global oscillations (Mayer waves) that are influenced by the sympatic nerve system and related to blood pressure fluctuations (Bumstead, Bauer, Wright, & Culver, 2016; Julien, 2006; Yücel et al., 2016) or vasomotion which has a spontaneous, local character (Aalkjaer, Boedtkjer, & Matchkov, 2011; Aalkjaer & Nilsson, 2005; Franceschini, Fantini, Toronov, Filiaci, & Gratton, 2000; Gratton et al., 1998). Normally, these SSHOs are seen as confounding signals and are removed before further analysis. Despite the fact that these SSHOs have been known for a long time (Jones, 1852), their precise origin and function is still unknown. If these oscillations were better understood, they could provide a window to certain physiological processes in the brain, and become valuable sources of information, rather than confounding factors in other measurements only.

With hyperspectral imaging [also called Multi‐Spectral Optical Intrinsic Signal Imaging (MS‐OISI)], images can be made at multiple wavelengths to calculate oxygenated and deoxygenated hemoglobin concentrations (Bouchard, Chen, Burgess, & Hillman, 2009; Hilman, 2007). This method can be used to directly observe SSHOs on the human cortex itself. Due to the invasive character of having to remove the skull and dura, direct SSHO observations have been only sporadically described (Mitra, Ogawa, Hu, & Uǧurbil, 1997; Obrig et al., 2000; Rayshubskiy et al., 2014).

In this article, we provide a thorough description of the observed SSHOs in four patients with epilepsy who underwent epilepsy surgery in the UMC Utrecht. Using Fourier analysis, we were able to produce highly detailed spatial information on the power and phase of oxygenated and deoxygenated hemoglobin SSHOs. These detailed SSHOs analyses may add to a better characterization and understanding of the SSHOs in the human brain.

## 2 MATERIALS AND METHODS

### 2.1 Patient characteristics and clinical evaluation for epilepsy surgery

We included four patients (2 males and 2 females, age on average 17 years, ± 4.5 years) who underwent epilepsy surgery between 2009 and 2012. The medical information of the patients is given in Table 1.

**Table 1:** Patient characteristics

Patient	Age	Gender	Pathology	Location camera	Imaging year
1	22	F	No clear abnormalities	Right, frontocentral	2009
2	19	M	FCD	Right, frontocentral	2012
3	13	F	DNET I	Left, frontal parasagittal	2012
4	13	M	TSC: FCD IIb	Left parietal	2012

Patients 1 and 2 underwent intracranial EEG monitoring during 3–7 days as pre‐surgical evaluation. During this clinical monitoring period, electrode grids were placed on the cortex to delineate the seizure onset zone and functional regions. Electrical stimulation mapping (ESM) was applied, in which 30–60 Hz cortical stimulation during 1–7 s was applied to a small area of cortex, while its effect on function was observed. The effect of the stimulation was recorded as either an instantaneous positive effect (e.g., an involuntary contraction of the thumb or any motor behavior), or a subjective experience (e.g., a tingling in a finger). This outcome was used to map functions on the cortex.

In patients 3 and 4, electrode grids were placed on the cortex only during surgery to delineate the epileptogenic region for resection. In patient 3, a Somatosensory Evoked Potential (SSEP) was applied during surgery and the central sulcus was located. With ESM, motor and sensory leg was located interhemispherically. No motor mapping was performed on visible cortex, so no motor and sensory hand/arm were located. In patient 4, no SSEP or ESM was performed.

### 2.2 Hyperspectral imaging

In all patients, hyperspectral recordings were made during surgery, while the surgeon was not operating. The recordings were approved by the Medical Ethical committee of the University Medical Center of Utrecht. All four patients gave informed consent to make hyperspectral recordings during surgery.

#### 2.2.1 Hyperspectral imaging system 1

**Figure 1:**
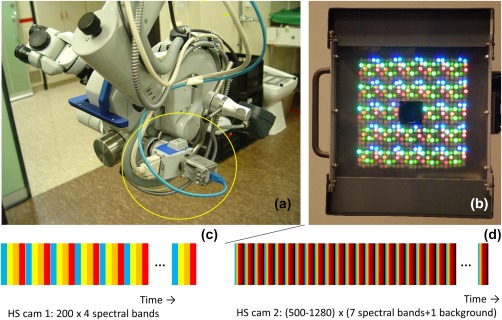
Hyperspectral camera systems used in imaging perfusion and oxygenation variations in human brain tissue. (a) High resolution monochrome camera in combination with a Liquid Tunable Filter attached to a surgical microscope. (b) A hyperspectral light source consisting of LEDs ranging from UV, VIS to NIR subsequently illuminating the brain surface. The high resolution camera is located at the center of the light source. Below the spectral bands C and D scanned by both systems. The color of the bands mimic the spectral bands, the bands’ size is related to the exposure time. The black bands in b indicate scans without illumination by the LED light source [Color figure can be viewed at http://wileyonlinelibrary.com]

Patient 1 was imaged in 2009 during grid explanation surgery, with a hyperspectral camera system to see whether changes in oxygenation could be perceived by the imaging system. This system consisted of a hyperspectral camera mounted to the assistant's tube of a surgical microscope (Figure 1a). It consisted of a liquid crystal tunable filter (Cri VariSpec VIS bandwidth (FWHM) 10 nm) and a monochrome camera with 1392 × 1024 resolution (PCO PixelFly QE) (Noordmans et al., 2013). A polarizer was placed in the incident path of the microscope light, cross‐polarized to the tunable filter, to suppress reflections from the surface.

The patient was continuously imaged for 7 min looping over 4 specific spectral bands (duration 0.46 s per spectral band). The imaging at this group of 4 spectral bands was repeated 200 times. The central wavelengths of the spectral bands were 480, 570, 600, and 660 nm, which were chosen such that the oxy‐ and deoxy‐absorption spectrum differed at these wavelengths and that the light was scattered at deeper tissue layers while still within the transmission range of the Cri filter. With this system oxygenation values could be determined once per 4 × 0.46 s = 1.8 s.

During the measurements, a seizure was captured. Measurements showed increase in blood flow during the seizure (Noordmans et al., 2013). In this study, we only included the interictal measurements.

#### 2.2.2 Hyperspectral imaging system 2

A second version hyperspectral system with hyperspectral light source and monochrome camera was used for patients 2,3, and 4 in 2012, in which detection of oxygenation values was four times faster, roughly 2 times per second.

This system consisted of a flat panel light source with a total of 600 LEDs with 17 different peak wavelengths (of which 7 were used in these experiments), with a CMOS camera mounted in the middle of the panel (BCi5 CMOS Camera, C‐Cam Technologies) (Figure 1b) (Klaessens, Nelisse, Verdaasdonk, & Noordmans, 2013). No polarization sheet was used. Patients were imaged for about 7 min with 500 to 1280 groups of 7 spectral bands (470 nm, 525 nm, 625 nm, 690 nm, 750 nm, 810 nm, and 850 nm, bandwidth (FWHM) 20–30 nm), duration 0.06‐0.09 s per spectral band. In every sweep along the 7 spectral bands, an image was made with LED light source switched off. By subtracting this directly, during acquisition, from the other spectral images, ambient light could be suppressed (Supporting Information Figure 1). As more wavelengths were captured, the absolute oxy‐ and deoxyhemoglobin concentrations could be calculated and, thus, the saturation values.

### 2.3 Acquisition parameters

The acquisition parameters for all patients are given in Table 2. It shows that with the second system, we could image faster and image more wavelengths within the same period of time. The increasing exposure time of the camera for patients 2 to 4 reflects increased optimization of the exposure time for a good signal while not overflowing the 8‐bit CMOS camera.

**Table 2:** Acquisition parameters

Patient	Resolution	HSsystem	Number ofwavelengths	Backgroundcorrection	Number ofwavelengthblocks	Exposuretime [ms]	Time perwavelengthblock [ms]	Total acquisitiontime [mins:secs]
1	696x512	1	4	no	200	379	1836	06:07
2	640x512	2	7	yes	500	4	515	04:17
3	640x512	2	7	yes	1280	15	487	10:24
4	640x512	2	7	yes	940	30	655	10:16

### 2.4 Post‐processing

For both systems, reflection (backscatter) images were calculated by dividing the acquired images by the spectrum from a white surgical tissue placed on the brain (visible during acquisition). Motion (translation and rotation) was compensated using custom written image registration software (Noordmans, van den Biesen, de Roode, & Verdaasdonk, 2008). The oxygenation and de‐oxygenation values were calculated by modeling the spectral absorption of the light traveling through the top layers of the brain tissue and scattered back to the camera. This was done by two different calculation methods, the Delta‐t method and the fit‐method (Klaessens et al., 2013) (See also Supporting Information Figure 2):

*Delta‐t method*: For all patients, we used the delta‐t method to get a strong signal of the changes in oxygenation ${\Delta c}_{HbO}$ and ${\Delta c}_{HbR}$. Using the reflection image $R(\vec{x},t,\lambda )$, (where $\vec{x}$ denotes position, t time, and λ wavelength) at t=0 s as a reference, the Delta‐t formula becomes:

(1) −log⁡(R(x→,t,λ10))=−log⁡(R(x→,0,λ10))+ΔcHbO(x→,t)⋅AHbO(λ)+ΔcHbR(x→,t)⋅AHbR(λ),

where $A_{HbO}(\lambda )$ and $A_{HbR}(\lambda )$ represent the absorption spectra of oxy‐hemoglobin and deoxy‐hemoglobin respectively. The change in concentrations were then calculated using least square fitting with pseudo inverse matrix calculations. The Delta‐*t* method results in a set of Δ *c*_HbO_ and Δ *c*_HbR_ images, where the change in HbO and HbR concentrations are known per pixel at each moment in time (with respect to the situation at t=0).

*Fit‐method*: When more than 4 wavelength images were captured along the spectral axis (patient 2–4), we could also calculate the absolute hemoglobin concentrations, the total hemoglobin HbT and saturation values. With this method, the reflection image could be decomposed into an oxygenation concentration $c_{HbO}$, a deoxygenation concentration $c_{HbR}$, and two (wavelength independent) constants $C_{1}(\vec{x},t)$ and $C_{2}(\vec{x},t)$.

(2) −log⁡(R(x→,t,λ10))=cHbO(x→,t)⋅AHbO(λ)+cHbR(x→,t)⋅AHbR(λ)+C1(x→,t)+C2(x→,t)⋅λ

The concentrations were then calculated using least square fitting with pseudo inverse matrix calculations. The total hemoglobin is then calculated by

(3) $$c_{HbT}\left(\vec{x},t\right)=c_{HbO}\left(\vec{x},t\right)+c_{HbR}\left(\vec{x},t\right),$$

and the saturation by

(4) $$sat\left(\vec{x},t\right)=\frac{c_{HbO}(\vec{x},t)}{c_{HbT}\left(\vec{x},t\right)},$$

The Fit‐method results in a set of *c*_HbO_ and *c*_HbR_ images, where the HbO and HbR concentrations are known per pixel at each moment in time.

### 2.5 Frequency analysis

To analyze the SSHOs, which were exceptionally stationary during the entire measurement period for all patients, a Fourier transform was performed for all HbO and HbR images, yielding amplitude and phase images along the temporal frequency axis (Supporting Information Figure 3). The Delta‐t method resulted in two amplitude image sets ${Im}_{Amp\;\Delta HbO}$ and ${Im}_{Amp\;\Delta HbR}$ and two phase image sets ${Im}_{Phase\;\Delta HbO}$ and ${Im}_{Phase\;\Delta HbR}$ for each patient. For patients 2–4, where we had captured images at a larger number of wavelengths, we could also use the Fit‐method resulting in two additional amplitude and phase images: ${Im}_{Amp\;HbO}$ and ${Im}_{Amp\;HbR}$and ${Im}_{Phase\;HbO}$ and ${Im}_{Phase\;HbR}$. Then, the amplitude images of Delta‐t HbO ( ${Im}_{Amp\;\Delta HbO}$) were inspected along the frequency axis to look for coherent regions which suddenly had a stronger amplitude at a certain frequency, resulting in SSHO‐regions. The reason to choose for the Delta‐t HbO signal here was that the Delta‐t method yielded a stronger signal than the Fit‐method and that the HbO signal appeared to be stronger than the HbR signal. These regions were manually segmented using a custom‐made drawing program written in Java (Java navigation), and subsequently numbered from lower to higher SSHO frequencies. For each SSHO‐region the following parameters were extracted:

- Maximal amplitude for the Delta‐t signal of HbO and HbR. This amplitude was compared to a noise value, which was taken as the average amplitude value within a circular area of 5 mm diameter without visible SSHO. From inspecting all images, it appeared that this noise value did not vary along the frequency axis, so we could determine it from an image at an arbitrary Fourier frequency.
- Total hemoglobin, average saturation and maximal saturation change (amplitude) within the region when there were more than four wavelength images to also use the Fit method (patient 2–4).
- Maximal phase difference ${\Delta \phi }_{region,\Delta HbO}$ found extracted from phase image ${Im}_{Phase\;\Delta HbO}$ within an SSHO region to see whether the SSHO is synchronous across a region. It is expressed as fraction of an oscillation period in seconds.
- Wavefront velocity images are calculated by inverting the gradient of the phase image ${Im}_{Phase\;\Delta HbO}$: $1/\sqrt{{\left(\frac{\partial ({Im}_{Phase\;\Delta HbO}(\vec{x}))}{\partial x}\right)}^{2}+{\left(\frac{\partial ({Im}_{Phase\;\Delta HbO}(\vec{x}))}{\partial y}\right)}^{2}}$. Statistics like minimal, median, average and maximal velocity are then determined for each region.
- The phase difference ${\Delta \phi }_{\Delta HbO-\Delta HbR}$ between the HbO and the HbR signal ( ${Im}_{Phase\;\Delta HbO}-{Im}_{Phase\;\Delta HbR}$) to see whether the HbO and HbR SSHOs are synchronous. As this phase difference appeared to be rather constant within a region, the values were averaged within the region for analysis. For stationary signals, this phase difference ${\Delta \phi }_{\Delta HbO-\Delta HbR}=-hPod$ defined by Watanabe et al. (2017).

To calculate the two phase differences ${\Delta \phi }_{region,\Delta HbO}$ and ${\Delta \phi }_{\Delta HbO-\Delta HbR}$ a 2D phase unwrap algorithm was needed to create a continuous phase image within each region as the phase was often wrapped around 2π in a region (Herráez, Burton, Lalor, & Gdeisat, 2002).

## 3 RESULTS

### 3.1 Heart rhythm and respiration

**Figure 2:**
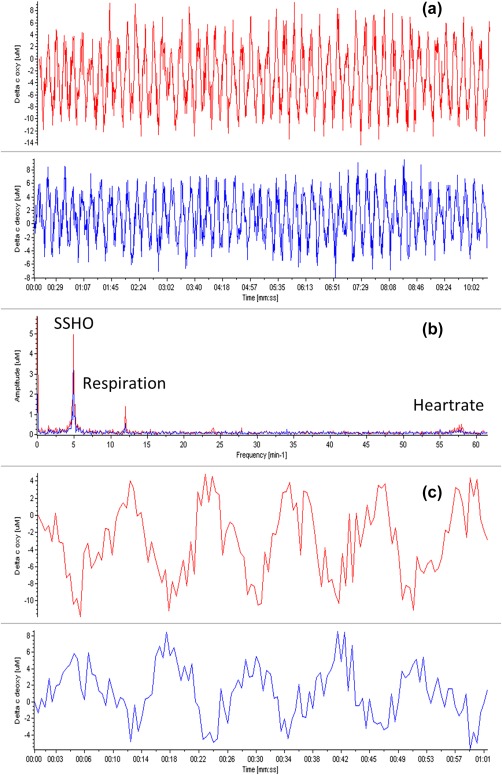
(a) Plots of changes in oxy (red) and deoxy (blue) concentrations of patient 3 at location 1 (indicated by the plus symbol in Figure 6E, near retractor). (b) Corresponding power spectra. Clearly peaks are seen for the SSHOs around 0.1 Hz, and the respiration and heart rate, the latter two corresponding to the rates reported by the anesthesia machine of 12 and 56 min^−1^, respectively. (c) Zooming on the curves of (a). A phase shift of about 180 degrees can clearly be seen between the changes in concentration of oxygenation and deoxygenation [Color figure can be viewed at http://wileyonlinelibrary.com]

For all patients, faster oscillations like respiration and sometimes heart rhythm were observed. The respiration rate of 12 to 18 min^−1^ corresponded to that supplied by the anesthesia machine. The heart rate (around 56 min^−1^) was only observed in patient 3 where the sampling rate of the hyperspectral camera was sufficiently high above the Nyquist criterion. This heart rate corresponded with the heart rate recorded by the anesthesia machine (Figure 2). Both the respiration and heartrate effects were seen across the entire exposed cortex, contrary to the local character of the SSHOs described next.

### 3.2 SSHOs

**Figure 3:**
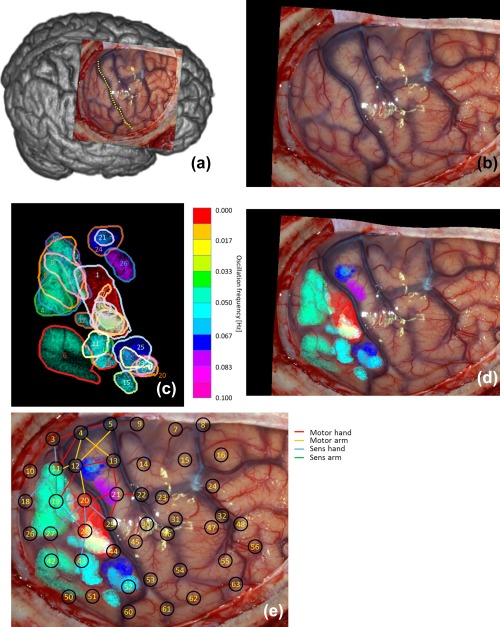
(a) Overview of observed brain and central sulcus as yellow dotted line of patient 1. (b) Photo of exposed brain. (c) Found 25 regions with SSHOs. Each color corresponds to a different SSHO frequency. (d) Regions overlain over original photo. (e) Locations intra‐cortical EEG grid electrode overlain over (d). The red area corresponds to the repetitive epileptic attack [Color figure can be viewed at http://wileyonlinelibrary.com]

**Figure 4:**
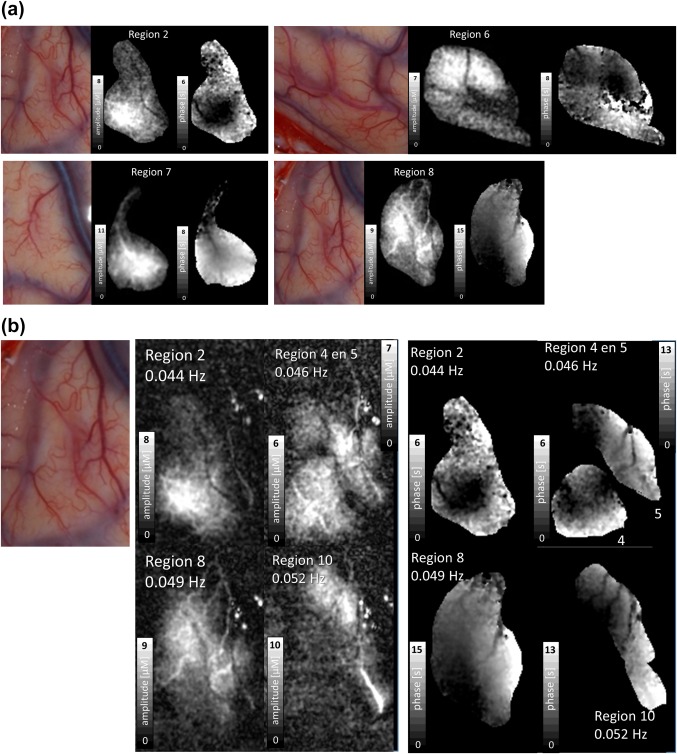
(a) Photo, amplitude and $\Delta \varphi_{region,\;\Delta HbO}$ phase images of four SSHO regions of patient 1. The images are intensity stretched individually to enhance details. See gray scales for exact values which can also be read from Table 3. Arteries do or just do not take part in the SSHO. The maximal phase difference within a region can approach one period, thus tens of seconds apart. (b) Amplitude and $\Delta \varphi_{region,\Delta \;HbO}$ phase images of four SSHO regions of patient 1 within the same area. The images are intensity stretched individually to enhance details. See gray scales for exact values which can also be read from Table 3. Note that SSHO regions can strongly overlap, seem to relate to individual blood vessels and that these vessels do not show up in another SSHO region [Color figure can be viewed at http://wileyonlinelibrary.com]

**Figure 5:**
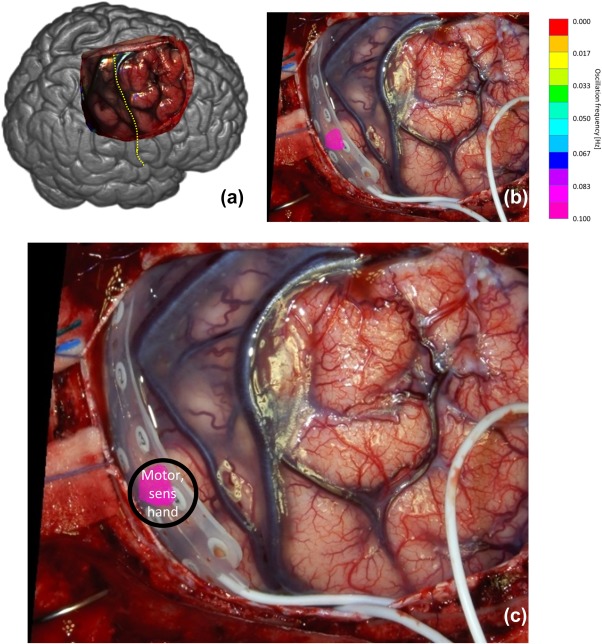
(a) Overview of observed brain and central sulcus as yellow dotted line of patient 2. (b) Photo of exposed brain. (c) SSHO region overlain of photo with intra‐cortical grid. Patient 2 only showed one oscillating area. Note that during multi‐spectral capturing the EEG grid was not present on the brain tissue [Color figure can be viewed at http://wileyonlinelibrary.com]

**Figure 6:**
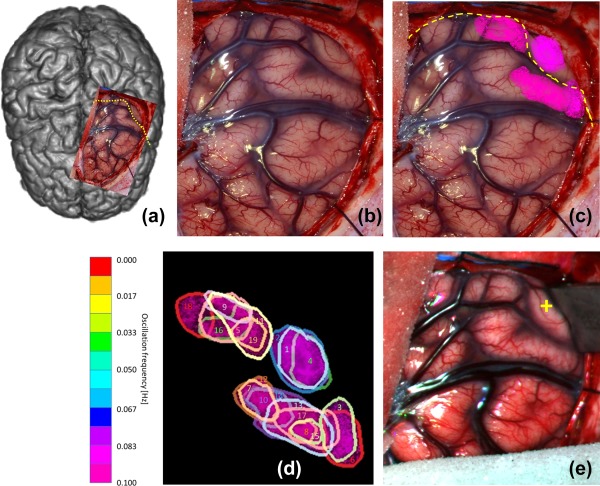
(a) Overview of observed brain and central sulcus as yellow dotted line of patient 3. (b) Photo of exposed brain. (c) SSHO regions overlain over photo. Each color corresponds to a different SSHO frequency. (d) Numbering of the 18 regions with SSHOs. (e) Color image reconstructed from hyperspectral recording showing the position of the retractor during acquisition [Color figure can be viewed at http://wileyonlinelibrary.com]

**Figure 7:**
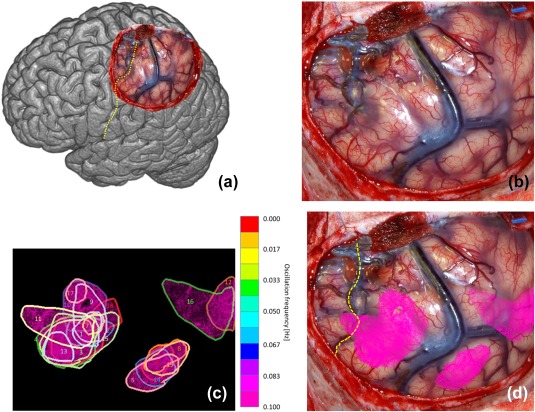
(a) Overview of observed brain and central sulcus as yellow dotted line of patient 4. (b) Photo of exposed brain. (c) Found 19 regions with SSHOs. Each color corresponds to a different SSHO frequency. (d) Regions overlain over original photo [Color figure can be viewed at http://wileyonlinelibrary.com]

In all patients, SSHOs were observed. Figures 3, 4, 5, 6, 7 show the amplitude of the SSHOs up to frequencies of about 0.1 Hz. Noise values remained the same across the image for all frequencies and were below 1µM for patients 1 and 2, and below 0.4µM for patients 3 and 4 (because of the longer exposure time). The SSHOs were constantly visible during the entire imaging duration of 4–10 min.

**Figure 8:**
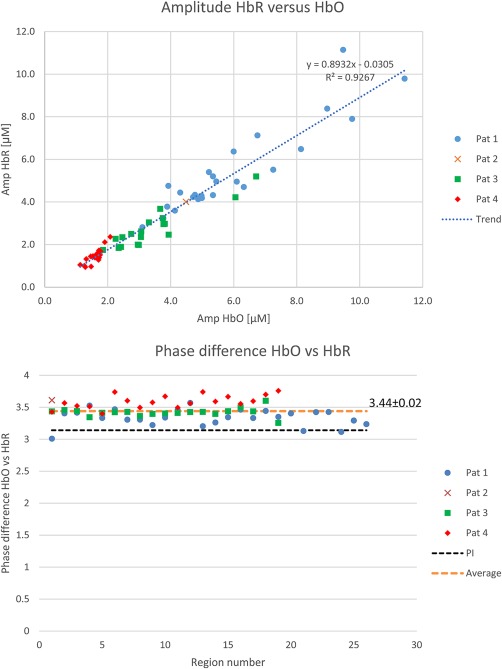
Top: Relation between HbR and HbO amplitude for all SSHOs (including trend based on linear regression). It demonstrates that the HbR amplitude is about 90% of the HbO amplitude. Bottom: phase difference $\Delta \varphi_{\Delta HbO-\Delta HbR}$ between HbR and HbO oscillation. It appears that the HbR oscillation lags slightly more than π behind the HbO oscillation [Color figure can be viewed at http://wileyonlinelibrary.com]

In patient 2, only one SSHO‐region was observed. In the other patients, many SSHO‐regions were visible. SSHOs were visible in distinct anatomical areas, although there were also large parts of the exposed cortex without visible SSHOs. Some areas contained a SSHO‐region with one frequency, others contained SSHO‐regions with multiple frequencies. For all SSHO‐regions, the amplitude of the HbR SSHO was 11–12% smaller than that of the HbO SSHO (Figure 8a). The HbR SSHO was always approximately in opposite phase compared to the HbO SSHO (Figure 8b), resulting in a much lower amplitude of the total hemoglobin (HbT = Hbr + HbO) SSHO. Videos showing the amplitude of the SSHOs are given as Supporting Information.

### 3.3 Location of SSHO regions

In patient 1, 25 distinct regions were seen with different SSHO frequencies (Figure 3, Table 3), ranging from 0.044 Hz to 0.082 Hz. Region 1 is omitted as it is actually not a SSHO, but corresponds to the region with increased perfusion only once during the image acquisition (Noordmans et al., 2013). Some regions did not overlap like region 6, 15 and 26, other SSHO‐regions did overlap (e.g. region 5, 8, 10). The HbO and HbR amplitudes differed per region from 3 to 11 µM. The maximal phase variation of HbO within a SSHO‐region $\Delta \phi_{region,\;\Delta HbO}$ could be quite large, for example 70% of a period for region 8. The amplitude and phase of the HbO SSHO within some particular regions are shown in Figure 4a. When zooming in on different SSHO‐regions with overlap (Figure 4b), one clearly sees that the small vessels take part only in one SSHO. They perfuse the same anatomical area but with another oscillating frequency.

**Table 3:** Statistics regions patient 1 (noise level max 1 µM)

				Max amp	Max amp	Max phase diff, $\Delta \varphi_{region,\;\Delta HbO}$	
Region	Freq [Hz]	Period [s]	Area [mm^2^]	HbO osc [µM]	HbR osc [µM]	Period [0–1]	Time [s]
1	0.005	183.6	142	9.8	7.9	0.24	45.5
2	0.044	22.9	98	8.1	6.5	0.25	5.9
3	0.046	21.6	18	4.7	4.2	0.24	5.4
4	0.046	21.6	55	6.1	5.0	0.29	6.3
5	0.046	21.6	59	6.8	7.1	0.58	12.8
6	0.049	20.4	129	7.3	5.5	0.39	8.1
7	0.049	20.4	49	11.4	9.8	0.40	8.3
8	0.049	20.4	134	9.0	8.4	0.71	14.9
9	0.052	19.3	16	6.0	6.4	0.47	9.3
10	0.052	19.3	71	9.5	11.1	0.65	12.8
11	0.054	18.4	28	6.3	4.7	0.35	6.6
12	0.054	18.4	14	4.1	3.6	0.19	3.5
13	0.054	18.4	13	4.3	4.4	0.27	5.1
14	0.054	18.4	24	4.9	4.1	0.37	6.9
15	0.057	17.5	18	5.0	4.2	0.27	4.9
16	0.057	17.5	24	5.3	4.3	0.41	7.3
17	0.060	16.7	14	5.0	4.3	0.23	4.0
18	0.060	16.7	12	5.2	5.4	0.36	6.1
19	0.060	16.7	33	3.8	3.2	0.34	5.8
20	0.063	16.0	20	4.8	4.3	0.20	3.3
21	0.063	16.0	11	3.9	4.8	0.23	3.8
22	0.065	15.3	38	4.9	4.2	0.69	10.8
23	0.068	14.7	19	5.4	5.0	0.24	3.5
24	0.071	14.1	44	5.3	5.2	0.41	6.0
25	0.074	13.6	55	3.1	2.8	0.61	8.5
26	0.082	12.2	36	3.9	3.8	0.43	5.4

These SSHO‐regions were still visible in second recording, when the hyperspectral camera was shortly left on during the first surgery actions. A 4x faster scan (4x shorter exposure times) was made for 2.5 min with many motion errors due to surgery. Despite these artefacts, no changes in SSHO‐regions or frequencies were visible in the area somewhat away from the incised tissue.

SSHO‐regions were mainly located in parietal cortex, thus somatosensory areas. Two small SSHO‐regions were found in frontal cortex, thus motor area. The SSHO‐regions in both parietal and frontal cortex overlapped with the area where hand and arm function were found with ESM.

Patient 2 only showed one SSHO‐region with a frequency of 0.093 Hz (Figure 4, Table 4). The SSHO's amplitude was 4.5 µM, which was in the same range as for patient 1. The change in saturation was 1.8 percentage points (pp) at a mean saturation value of 56%. The saturation in other parts of the exposed brain without SSHO‐regions was a bit higher: 70% on the average.

**Table 4:** Statistics regions patient 2 (noise level max 1 µM)

Region	Freq [Hz]	Period [s]	Area [mm^2^]	Max amp HbO osc [µM]	Max amp HbR osc [µM]	HbT [µM]	$\bar{sat}$ [%]	Δsat [%]	Max phase diff, $\Delta \varphi_{region,\Delta \;HbO}$	
									Period [0–1]	Time [s]
1	0.093	10.7	36	4.5	4.0	114	56	1.8	0.19	2.0

With ESM, one area was found with both motor and sensory hand. This area was located in the parietal cortex and overlapped exactly with the SSHO‐region that was found with the spectral camera.

Patient 3 showed 18 different SSHO‐regions (Figure 5, Table 5) in the area of trepanation. The frequencies of the SSHOs ranged from 0.079 to 0.101 Hz. The change in saturation, obtained from the absolute oxy and deoxyhemoglobin concentrations, ranged from 1.6 to 5.2 pp. The saturation within the SSHO‐regions was 54% on average, while it was 65% in other parts of the brain without SSHO‐regions. In this patient, no ESM was performed. The SSHO‐regions seemed to be located on the parietal sensory hand area and frontal motor hand area.

**Table 5:** Statistics regions patient 3 (noise level max 0.4 µM)

Region	Freq [Hz]	Period [s]	Area [mm^2^]	Max amp HbO osc [µM]	Max amp HbR osc [µM]	HbT [µM]	$\bar{sat}$ [%]	Δsat [%]	Max phase diff, $\Delta \varphi_{region,\;\Delta HbO}$	
									Period [0–1]	Time [s]
1	0.079	12.7	42	2.4	1.9	115	63	1.9	0.18	2.2
2	0.082	12.2	57	6.7	5.2	115	63	5.2	0.21	2.4
3	0.083	12.0	33	3.8	3.0	149	57	2.3	0.23	2.6
4	0.085	11.8	31	2.3	1.8	114	65	1.8	0.19	2.2
5	0.087	11.6	39	1.9	1.7	123	54	1.5	0.22	2.4
6	0.087	11.6	34	3.3	3.0	181	61	1.7	0.20	2.2
7	0.088	11.3	19	3.0	2.0	145	57	1.7	0.16	1.8
8	0.088	11.3	8	2.5	2.3	150	63	1.6	0.16	1.8
9	0.090	11.1	14	3.8	3.2	128	53	2.7	0.29	3.1
10	0.090	11.1	69	3.8	3.0	141	58	2.4	0.28	3.0
11	0.091	10.9	44	3.7	3.7	126	43	2.9	0.24	2.6
12	0.091	10.9	26	3.1	2.6	150	56	1.9	0.13	1.4
13	0.093	10.8	52	6.1	4.2	139	59	3.7	0.28	2.9
14	0.095	10.6	23	3.0	2.0	135	60	1.8	0.15	1.6
15	0.095	10.6	9	2.7	2.5	139	63	1.9	0.11	1.1
16	0.096	10.4	21	3.9	2.5	119	54	2.7	0.30	3.0
17	0.096	10.4	37	3.1	2.4	138	61	2.0	0.18	1.8
18	0.101	9.9	24	2.3	2.3	141	47	1.6	0.43	4.1

Patient 4 showed 19 different SSHO‐regions (Figure 6, Table 6), spread across the entire area of the resection. The frequencies of the SSHOs ranged from 0.065 to 0.119 Hz. The change in saturation ranged from 0.8 pp to 2.2 pp (region 9). The saturation in the SSHO‐regions was 61% on the average, and 63% in the brain without SSHO‐regions.

**Table 6:** Statistics regions patient 4 (noise level max 0.4 µM)

Region	Freq [Hz]	Period [s]	Area [mm^2^]	Max amp HbO osc [µM]	Max amp HbR osc [µM]	HbT [µM]	$\bar{sat}$ [%]	Δsat [%]	Max phase diff, $\Delta \varphi_{region,\;\Delta HbO}$	
	Period [0–1]								Time [s]
1	0.065	15.4	32	1.3	1.0	125	73	0.9	0.14	2.1
2	0.071	14.0	49	1.3	0.9	117	72	1.0	0.20	2.7
3	0.076	13.1	97	1.6	1.5	154	73	1.0	0.22	2.8
4	0.078	12.8	111	1.5	1.4	157	72	0.9	0.31	3.8
5	0.080	12.6	58	1.5	1.5	176	67	0.8	0.40	4.9
6	0.089	11.2	32	1.3	1.3	126	70	1.0	0.17	1.8
7	0.089	11.2	13	1.5	1.0	114	73	1.1	0.11	1.2
8	0.096	10.4	37	1.7	1.3	122	69	1.2	0.16	1.6
9	0.096	10.4	69	2.1	2.4	100	64	2.2	0.28	2.8
10	0.099	10.1	86	1.7	1.6	99	65	1.7	0.28	2.8
11	0.102	9.8	99	1.7	1.4	136	65	1.1	0.39	3.7
12	0.104	9.6	53	1.7	1.7	147	60	1.2	0.29	2.7
13	0.109	9.2	30	1.6	1.4	169	77	0.9	0.20	1.8
14	0.110	9.1	48	1.8	1.5	150	68	1.1	0.23	2.0
15	0.112	8.9	145	1.7	1.7	141	69	1.2	0.28	2.4
16	0.114	8.8	121	1.9	2.1	123	48	1.6	0.46	3.9
17	0.115	8.7	16	1.1	1.0	103	71	1.1	0.25	2.1
18	0.117	8.5	118	1.5	1.4	141	69	1.0	0.23	1.9
19	0.119	8.4	42	1.8	1.7	141	69	1.2	0.17	1.4

In this patient, no ESM was performed. Based on the location of central sulcus, part of the SSHO‐region seemed to be anatomically located on the sensory hand area (parietal cortex).

**Figure 9:**
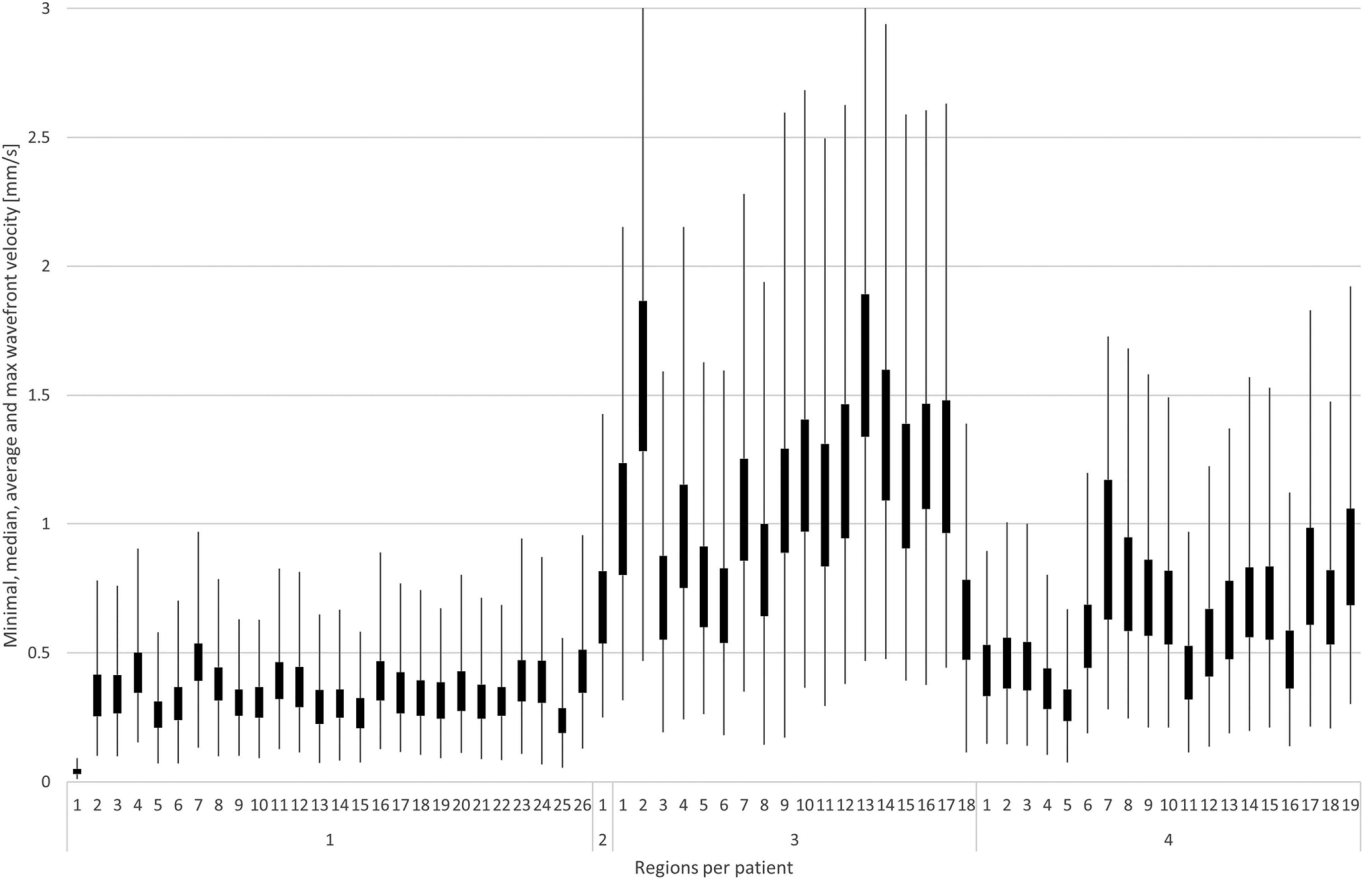
Wavefront velocity statistics for each SSHO based on ${Im}_{Phase\;\Delta HbO}$ phase images. For each region, the minimum velocity is given by the lowest point of the lowest line, the median velocity by the box bottom, the average velocity by the box top and the maximum velocity by the highest point of the highest line (clipped to zoom in on average and median velocity). Note that the wavefront velocity of patient 1 was almost a factor 2 lower than for the other patients

The observed SSHO phase difference within a region could be explained by a propagating hemodynamic wave. The wavefront velocity statistics for all regions and patients are shown in Figure 9. The SSHO wavefront velocity across all patients was found to be: average value 0.8 mm/s (0.4, 0.8, 1.3, 0.7 mm/s respectively for patients 1–4) and median value 0.5 mm/s (0.3, 0.5, 0.9, 0.5 mm/s).

## 4 DISCUSSION

SSHOs were observed in the oxy‐ and deoxyhemoglobin concentration variations on the cortical surface obtained with intra‐operative hyperspectral imaging in four patients with epilepsy. We observed SSHO‐regions with different specific frequencies that often showed spatial overlap. The phase shift between oxy‐ and deoxyhemoglobin SSHOs $\Delta \phi_{\Delta HbO-\Delta HbR}$ was about half an oscillation period, i.e. almost 180°. On average, the amplitude of the deoxyhemoglobin SSHOs was 11% smaller than that of oxyhemoglobin SSHOs. The maximal phase variation within a SSHO‐region $\Delta \phi_{region,\;\Delta HbO}$ was in the order of a few seconds but sometimes as large as 15 s. When associating a propagating wave to the phase variation within a SSHO region we found wavefront velocities in the order of 1 mm/s. Interestingly, these velocities are in line with capillary blood velocities observed in rodents and may indicate that SSHOs embody a local propagating hemodynamic wave, possibly related to vasomotion and maintenance of adequate tissue perfusion.

## 5 FINDINGS

The major findings in this article are:

### 5.1 Oscillating frequencies are stationary over time and have a very small bandwidth

Instead of applying a relatively wide bandpass filter, where all oscillations are aggregated (Bumstead et al., 2016; Gratton et al., 1998; Zuo et al., 2010), or only looking at only one frequency of 0.1 Hz (Rayshubskiy et al., 2014), a more detailed view on a SSHO can be acquired using a very small bandwidth ( Δf around 0.002 Hz) as with a Fourier transform. In this way, it becomes clear that different but also overlapping SSHO‐regions can be observed with slightly different SSHO frequencies.

### 5.2 Strong HbR oscillation

In contrast to Rayshubskiy et al (Rayshubskiy et al., 2014) who did not find strong HbR oscillations, we see strong HbR oscillations for all 4 patients (Figure 8). On average, the amplitude of the HbR SSHO is only 11% smaller than that of HbO SSHO. In agreement with others (Reinhard et al., 2006; Watanabe et al., 2017), we found that the HbR SSHO were almost out‐of‐phase with the HbO SSHO, $\Delta \phi_{\Delta HbO-\Delta HbR}∼\pi$. Therefore, the SSHO in total hemoglobin HbT (sum of HbO and HbR) is relatively small. Apparently, oxygen is periodically extracted from the blood vessels. The oscillations are predominantly cerebral oxygen exchange (CBE) oscillations (Watanabe et al., 2017; Wylie et al., 2009).

### 5.3 Continuous phase variation within a SSHO region may indicate a local propagating hemodynamic wave

Instead of cross‐correlating signals to image wave propagation (Rayshubskiy et al., 2014), we used the phase part of the Fourier transform to estimate the SSHO phase variation within a region. The phase was continuous within a SSHO‐region, indicating that the phase can be determined robustly across the region. Within a SSHO‐region, locations seem to be strongly connected; there are no locations with a totally different phase signal. It is unclear whether this connection has a direction, i.e. that the SSHO starts at one point and then propagates as a wave across the region or that it is just one oscillating network influencing each other. The notion of a propagating hemodynamic wave, however, can be substantiated by the observation that the estimated wavefront velocities are in line with capillary blood velocities in rodents, i.e. in the order of 1 mm/s (Stefanovic et al., 2008).

### 5.4 Oscillating frequencies differ between patients

While for patient 2 to 4 the oscillating frequencies lay between 0.08 and 0.12 Hz, the frequencies for patient 1 were lower, between 0.04 and 0.08 Hz. Retrospectively, technical tests were performed on software from 2009 and 2012 to confirm that the timings were correct. Anaesthetics appeared not to be that different that it could explain the difference in oscillation frequency. Differences in pathology, stress and location of resection could play a role, but we could not find such clues why the SSHO frequencies for patient 1 differed almost a factor two from the SSHO frequencies of patient 2 to 4.

### 5.5 SSHOs are found in local regions

It is striking that the SSHO‐regions are localized to specific anatomical areas. For patient 1, 2 and 3 the regions are located near the motor and somatosensory strip, for patient 4 around the more posterior part of the parietal area. Why the SSHO‐regions were only found there, remains unclear.

In patient 4, one SSHO‐region is located on the parietal cortex, outside the somatosensory area. The other three patients only showed SSHO‐regions in the somatosensory or motor area. The more posterior parietal cortex was not visible. Therefore, we do not know whether SSHO‐regions would also have been present in the parietal cortex in patient 1–3. The observation of the spectral camera should be repeated in more patients with visible parietal cortex during surgery.

## 6 FUTURE DIRECTIONS

Although our analysis gives new insight in the characteristics of SSHOs (distinct regions and various stable frequencies), several questions remain to be answered:

1. It has been hypothesized that SSHOs are linked to pathology (Rayshubskiy et al., 2014) and that tissue in stress can produce SSHOs. We think that stress may indeed play a role, but it is a factor that is difficult to measure. Mechanical stress could play a role, but if it would, one would expect to see SSHOs mainly at the edges of the skull opening, or more widespread over the entire exposed brain, while we see very focal regions. There might be a relation with the lower saturation values found within the SSHO‐regions. This was certainly the case for patient 2 and 3, but for patient 4 the saturation within the SSHO‐regions was only slightly smaller than outside these regions.
2. Another hypothesis is that the SSHOs are a spontaneous action of small vessels that play no physiological role (C. Aalkjaer et al., 2011; Franceschini et al., 2000; Hudetz, Biswal, Shen, Lauer, & Kampine, 1998; Julien, 2006; V. J. Kiviniemi et al., 2005; Mitra et al., 1997; Stefanovska, 2006; Yücel et al., 2016) (C. Aalkjaer et al., 2011; Franceschini et al., 2000; Hudetz et al., 1998; Julien, 2006; V. Kiviniemi, Kantola, Jauhiainen, Hyvärinen, & Tervonen, 2003; Mitra et al., 1997; Stefanovska, 2006; Yücel et al., 2016). However, it seems doubtful that the brain is ‘wasting energy’ in producing such oscillations (Fox & Raichle, 2007). SSHOs may rather be related to active functional areas where perfusion is critical and SSHOs reflect vasomotion, generated to improve perfusion (C. Aalkjaer et al., 2011). By avoiding a ‘latch state’ the small vessels are ready to increase their performance in perfusing the brain tissue. The brain tissue is very active, and it is important that the blood perfusion is ready to improve its capacity.
3. Several models have been proposed to describe hemodynamic changes in terms of arterial, capillary and venous compartments. Although they explain the BOLD response (Boas, Jones, Devor, Huppert, & Dale, 2008) and (slow) oscillations at a coarse level (Fantini, 2014), they do not explain the characteristics of the slow oscillations we found in our measurements. Our measurements confirm the phase difference of 1 π between the oxy and deoxy oscillation found by others (Reinhard et al., 2006; Watanabe et al., 2017). These models lack an explanation for the local (sometimes overlapping) character of the slow oscillations, its stationary aspect, their slightly different oscillation frequencies and the phase shift across the oscillating region. Our direct measurements of SSHOs on the human cortex are a clear incentive for further improving the hemodynamic models.

## 7 IMPLICATIONS FOR OTHER DOMAINS

### 7.1 Near‐infrared spectroscopy

As we observed SSHOs in all patients, SSHOs can be detected using NIRS (Obrig et al., 2000; Sakudo, 2016), also when a patient is sedated. A SSHO may be confused with Mayer waves that are known to be globally present in all vessels. In our opinion, as we did not see any global oscillation across the brain cortex, global Mayer waves can occur more focal than normally described. Studies in patients that are awakened during surgery, for example, to determine the speech areas for functional neurosurgery, may reveal whether SSHOs change their characteristics during sedation.

### 7.2 (resting state) functional MRI

As functional magnetic resonance imaging looks at changes in deoxygenated blood levels, it is sensitive to the HbR SSHOs. Such oscillations have been observed in fMRI signals (Tong & Frederick, 2010). It can therefore be advantageous to include the continuous oscillating aspect of SSHOs in interpreting the BOLD signal. By modelling an oscillating component, one might increase the sensitivity of the BOLD fMRI analysis.

In the analysis of resting state functional MRI, correlations are found in the BOLD signal between brain areas across the entire brain (Fox & Raichle, 2007), producing the so called resting‐state networks such as the default mode network. Knowing that SSHOs exist, having very steady frequency and a local character, one might model and include this in the analysis to identify resting state networks within brain regions, i.e. at a much smaller spatial scale than previously studied in resting‐state fMRI.

## 8 LIMITATIONS

### 8.1 Scattering

Note that scattering has been ignored in these calculations. We acknowledge that it should be included for correct estimation of hemoglobin concentrations. However, it is unclear how scattering should be included for reflection (backscatter) imaging of the human brain. Most values are derived from bloodless ex‐vivo brain tissue, while blood will be the major absorber and prevent light to be scattered and thus reach the penetration depth as specified by literature (Sterenborg, Gemert, Kamphorst, Wolbers, & Hogervorst, 1989). To get an idea on possible impact, we recalculated the hemoglobin concentration when assuming a three times higher penetration depth at 850 nm compared to 470 nm. The hemoglobin concentration then became 40% lower. Furthermore, this will only affect the absolute concentration and saturation values. It does not affect the amplitudes of the HbO, HbR and thus saturation SSHOs.

### 8.2 Region selection

The selection of the SSHO‐regions by hand can be considered arbitrary. Although the strongest oscillations were easy to recognize, there were often regions that oscillated weakly. It is hard to devise an algorithm to automatically extract the regions as the boundaries are difficult to define when the oscillating amplitude approaches that of the ‘noise level’. Also, larger vessels produce artefacts (probably due to motion) having strong power at the oscillating frequency, preventing the use of an easy thresholding algorithm.

### 8.3 Time analysis

As the SSHOs appeared to be exceptionally stationary, Fourier analysis could be used to determine the amplitude and phase of the sinusoidal oscillations. When this would not be the case, e.g. when the SSHOs vanish and reappear, more advanced methods like wavelet, spectrogram or Hilbert transforms might be needed to study this dynamic behaviour (Watanabe et al., 2017).

## 9 CONCLUSIONS

SSHOs in oxygenation and deoxygenation could be found in all 4 patients with epilepsy. As the SSHOs are very stable over time, a Fourier transform can be used to study spatial amplitude and phase patterns of these SSHOs. It appears that many SSHO‐regions with slightly different frequencies can be seen on the cortex. Often SSHO‐regions do overlap giving a clear view on the individual vascular networks perfusing brain tissue. Hyperspectral imaging of the human cortex during surgery allow in‐depth characterization of SSHOs, and may give further insight in the nature and potential (clinical) use of SSHOs.

## Conflict of Interest

The authors report no conflicts of interests. The authors are supported with grants (ERC/epilepsy).

## Supporting information

Additional Supporting Information may be found online in the supporting information tab for this article.

Supporting Information

Supporting Information

Supporting Information

Supporting Information

Supporting Information

Supporting Information

Supporting Information

Supporting Information

## Footnotes

1 Alternative abbreviations for the same phenomenon are HONS (hemodynamic oscillations with narrow spectral width) (Bumstead et al., 2016) , LFO (low frequency oscillations) (Hocke, Tong, Lindsey, & de B. Frederick, 2016; Obrig et al., 2000; Zuo et al., 2010), MUSO (Monochromatic Ultra‐Slow Oscillations) (Nikulin et al., 2014), VLF (very low frequency) fluctuations (Kiviniemi et al., 2005), or VLFO (very low frequency oscillation) (Obrig et al., 2000).

## References

[hbm24194-bib-0001] Aalkjaer, C. , Boedtkjer, D. , & Matchkov, V. (2011). Vasomotion—what is currently thought? Acta Physiologica, 202(3), 253–269. doi:http://10.1111/j.1748-1716.2011.02320.x 21518271 10.1111/j.1748-1716.2011.02320.x

[hbm24194-bib-0002] Aalkjaer, C. , & Nilsson, H. (2005). Vasomotion: cellular background for the oscillator and for the synchronization of smooth muscle cells. British Journal of Pharmacology, 144(5), 605–616. doi:http://10.1038/sj.bjp.0706084 15678091 10.1038/sj.bjp.0706084 PMC1576043

[hbm24194-bib-0003] Boas, D. A. , Jones, S. R. , Devor, A. , Huppert, T. J. , & Dale, A. M. (2008). A vascular anatomical network model of the spatio‐temporal response to brain activation. NeuroImage, 40(3), 1116–1129. doi:http://10.1016/j.neuroimage.2007.12.061 18289880 10.1016/j.neuroimage.2007.12.061 PMC2577617

[hbm24194-bib-0033] Bouchard, M. B. , Chen, B. R. , Burgess, S. A. , & Hillman, E. M. C. (2009). Ultra‐fast multispectral optical imaging of cortical oxygenation, blood flow, and intracellular calcium dynamics. Optics Express, 17(18), 15670–15678. http://www.ncbi.nlm.nih.gov/pubmed/19724566 19724566 10.1364/OE.17.015670 PMC2760073

[hbm24194-bib-0004] Bumstead, J. R. , Bauer, A. Q. , Wright, P. W. , & Culver, J. P. (2016). Cerebral functional connectivity and Mayer waves in mice: Phenomena and separability. Journal of Cerebral Blood Flow & Metabolism, 37(2), 471. doi:http://10.1177/0271678X16629977 26868180 10.1177/0271678X16629977 PMC5381445

[hbm24194-bib-0005] Fantini, S. (2014). Dynamic model for the tissue concentration and oxygen saturation of hemoglobin in relation to blood volume, flow velocity, and oxygen consumption: Implications for functional neuroimaging and coherent hemodynamics spectroscopy (CHS). NeuroImage, 85, 202–221. doi:http://10.1016/j.neuroimage.2013.03.065 23583744 10.1016/j.neuroimage.2013.03.065 PMC3760999

[hbm24194-bib-0006] Fox, M. D. , & Raichle, M. E. (2007). Spontaneous fluctuations in brain activity observed with functional magnetic resonance imaging. Nature Reviews Neuroscience, 8(9), 700–711. doi:http://10.1038/nrn2201 17704812 10.1038/nrn2201

[hbm24194-bib-0007] Franceschini, M. A. , Fantini, S. , Toronov, V. , Filiaci, M. E. , & Gratton, E. (2000). Cerebral hemodynamics measured by near‐infrared spectroscopy at rest and during motor activation. Proceedings Qof the Optical Society of America in Vivo Optical Imaging Workshop, 73–80. Retrieved from http://www.nmr.mgh.harvard.edu/PMI/people/mari/papers/NIH99 mari.pdf

[hbm24194-bib-0008] Gratton, R. J. , Gandley, R. E. , McCarthy, J. F. , Michaluk, W. K. , Slinker, B. K. , & McLaughlin, M. K. (1998). Contribution of vasomotion to vascular resistance: a comparison of arteries from virgin and pregnant rats. Journal of Applied Physiology (Bethesda, Md. : 1985), 85(6), 2255–2260. Retrieved from http://www.ncbi.nlm.nih.gov/pubmed/9843550 10.1152/jappl.1998.85.6.2255 9843550

[hbm24194-bib-0009] Herráez, M. A. , Burton, D. R. , Lalor, M. J. , & Gdeisat, M. A. (2002). Fast two‐dimensional phase‐unwrapping algorithm based on sorting by reliability following a noncontinuous path. Applied Optics, 41(35), 7437–7444. doi:http://10.1364/AO.41.007437 12502301 10.1364/ao.41.007437

[hbm24194-bib-0034] Hilman, E. M. C. (2007). Optical brain imaging in vivo: techniques and applications from animal to man. J Biomed Opt, 12(5), 51402 10.1038/jid.2014.371 PMC2435254 17994863

[hbm24194-bib-0010] Hocke, L. M. , Tong, Y. , Lindsey, K. P. , & de B. Frederick, B. (2016). Comparison of peripheral near‐infrared spectroscopy low‐frequency oscillations to other denoising methods in resting state functional MRI with ultrahigh temporal resolution. Magnetic Resonance in Medicine, 76(6), 1697–1707. doi:http://10.1002/mrm.26038 26854203 10.1002/mrm.26038 PMC5796666

[hbm24194-bib-0011] Hudetz, A. G. , Biswal, B. B. , Shen, H. , Lauer, K. K. , & Kampine, J. P. (1998). Spontaneous fluctuations in cerebral oxygen supply. An introduction. Advances in Experimental Medicine and Biology, 454, 551–559. Retrieved from http://www.ncbi.nlm.nih.gov/pubmed/9889935 9889935 10.1007/978-1-4615-4863-8_66

[hbm24194-bib-0012] Jones, T. (1852). Discovery that veins of the bat's wing are endowed with rhythmical contractility and that the onward flow of blood is accelerated by each contraction. Philosophical Transactions of the Royal Society of London, 142(0), 131–136.

[hbm24194-bib-0013] Julien, C. (2006). The enigma of Mayer waves: Facts and models. Cardiovascular Research, 70(1), 12–21. doi:http://10.1016/j.cardiores.2005.11.008 16360130 10.1016/j.cardiores.2005.11.008

[hbm24194-bib-0014] Kiviniemi, V. J. , Haanpää, H. , Kantola, J.‐H. , Jauhiainen, J. , Vainionpää, V. , Alahuhta, S. , & Tervonen, O. (2005). Midazolam sedation increases fluctuation and synchrony of the resting brain BOLD signal. Magnetic Resonance Imaging, 23(4), 531–537. doi:http://10.1016/j.mri.2005.02.009 15919598 10.1016/j.mri.2005.02.009

[hbm24194-bib-0015] Kiviniemi, V. , Kantola, J. H. , Jauhiainen, J. , Hyvärinen, A. , & Tervonen, O. (2003). Independent component analysis of nondeterministic fMRI signal sources. NeuroImage, 19(2), 253–260. doi:http://10.1016/S1053-8119(03)00097‐1 12814576 10.1016/s1053-8119(03)00097-1

[hbm24194-bib-0016] Klaessens, J. H. G. M. , Nelisse, M. , Verdaasdonk, R. M. , & Noordmans, H. J. (2013). Multimodal tissue perfusion imaging using multi‐spectral and thermographic imaging systems, applied on clinical data. In *Proceedings of Multimodal Biomedical Engineering VIII* (Vol. 8574, pp. 1–8). doi:http://10.1117/12.2003823

[hbm24194-bib-0017] Mitra, P. P. , Ogawa, S. , Hu, X. , & UšUrbil, K. (1997). The nature of spatiotemporal changes in cerebral hemodynamics as manifested in functional magnetic resonance imaging. Magnetic Resonance in Medicine, 37(4), 511–518. doi:http://10.1002/mrm.1910370407 9094072 10.1002/mrm.1910370407

[hbm24194-bib-0018] Nikulin, V. V. , Fedele, T. , Mehnert, J. , Lipp, A. , Noack, C. , Steinbrink, J. , & Curio, G. (2014). Monochromatic Ultra‐Slow (∼0.1Hz) Oscillations in the human electroencephalogram and their relation to hemodynamics. NeuroImage, 97, 71. doi:http://10.1016/j.neuroimage.2014.04.008 24732648 10.1016/j.neuroimage.2014.04.008

[hbm24194-bib-0019] Noordmans, H. J. , Ferrier, C. , de Roode, R. , Leijten, F. , van Rijen, P. , Gosselaar, P. , … Verdaasdonk, R. (2013). Imaging the seizure during surgery with a hyperspectral camera. Epilepsia, 54(11), e150–e154. doi:http://10.1111/epi.12386 24199829 10.1111/epi.12386

[hbm24194-bib-0020] Noordmans, H. J. , van den Biesen, P. , de Roode, R. , & Verdaasdonk, R. (2008). Software helps see the unseen in ophthalmology. Laser Focus World, 44(5), Retrieved from http://www.laserfocusworld.com/articles/print/volume-44/issue-5/columns/software-computing/software-helps-see-the-unseen-in-ophthalmology.html

[hbm24194-bib-0021] Obrig, H. , Neufang, M. , Wenzel, R. , Kohl, M. , Steinbrink, J. , Einhaupl, K. , … Villringer, A. (2000). Spontaneous low frequency oscillations of cerebral hemodynamics and metabolism in human adults [In Process Citation]. Neuroimage, 12(6), 623–639. doi:http://10.1006/nimg.2000.0657 11112395 10.1006/nimg.2000.0657

[hbm24194-bib-0022] Rayshubskiy, A. , Wojtasiewicz, T. J. , Mikell, C. B. , Bouchard, M. B. , Timerman, D. , Youngerman, B. E. , … Hillman, E. M. C. (2014). Direct, intraoperative observation of ∼0.1Hz hemodynamic oscillations in awake human cortex: Implications for fMRI. NeuroImage, 87, 323–331. doi:http://10.1016/j.neuroimage.2013.10.044 24185013 10.1016/j.neuroimage.2013.10.044 PMC3961585

[hbm24194-bib-0023] Reinhard, M. , Wehrle‐Wieland, E. , Grabiak, D. , Roth, M. , Guschlbauer, B. , Timmer, J. , … Hetzel, A. (2006). Oscillatory cerebral hemodynamics‐the macro‐ vs. microvascular level. Journal of the Neurological Sciences, 250(1–2), 103–109. doi:http://10.1016/j.jns.2006.07.011 17011584 10.1016/j.jns.2006.07.011

[hbm24194-bib-0024] Sakudo, A. (2016). Near‐infrared spectroscopy for medical applications: Current status and future perspectives. Clinica Chimica Acta, 455, 181–188. doi:http://10.1016/j.cca.2016.02.009 10.1016/j.cca.2016.02.009 26877058

[hbm24194-bib-0025] Stefanovic, B. , Hutchinson, E. , Yakovleva, V. , Schram, V. , Russell, J. T. , Belluscio, L. , … Silva, A. C. (2008). Functional reactivity of cerebral capillaries. Journal of Cerebral Blood Flow & Metabolism, 28(5), 961–972. doi:http://10.1038/sj.jcbfm.9600590 18059431 10.1038/sj.jcbfm.9600590 PMC3197804

[hbm24194-bib-0026] Stefanovska, A. (2006). Coupled oscillators: complex but not complicated cardiovascular and brain interactions. *Conference Proceedings : … Annual International Conference of the IEEE Engineering in Medicine and Biology Society. IEEE Engineering in Medicine and Biology Society. Annual Conference*, 1(6), 437–40. doi:http://10.1109/IEMBS.2006.259557 10.1109/IEMBS.2006.259557 17946833

[hbm24194-bib-0027] Sterenborg, H. J. C. M. , Gemert, M. J. C. , Kamphorst, W. , Wolbers, J. G. , & Hogervorst, W. (1989). The spectral dependence of the optical properties of human brain. Lasers in Medical Science, 4(4), 221–227. doi:http://10.1007/BF02032451

[hbm24194-bib-0028] Tong, Y. , & Frederick, B. dB. (2010). Time lag dependent multimodal processing of concurrent fMRI and near‐infrared spectroscopy (NIRS) data suggests a global circulatory origin for low‐frequency oscillation signals in human brain. NeuroImage, 53(2), 553–564. doi:http://10.1016/j.neuroimage.2010.06.049 20600975 10.1016/j.neuroimage.2010.06.049 PMC3133965

[hbm24194-bib-0029] Watanabe, H. , Shitara, Y. , Aoki, Y. , Inoue, T. , Tsuchida, S. , Takahashi, N. , & Taga, G. (2017). Hemoglobin phase of oxygenation and deoxygenation in early brain development measured using fNIRS. Proceedings of the National Academy of Sciences, 114(9), E1737–E1744. doi:http://10.1073/pnas.1616866114 10.1073/pnas.1616866114 PMC5338505 28196885

[hbm24194-bib-0030] Wylie, G. R. , Graber, H. L. , Voelbel, G. T. , Kohl, A. D. , DeLuca, J. , Pei, Y. , … Barbour, R. L. (2009). Using co‐variations in the Hb signal to detect visual activation: A near infrared spectroscopic imaging study. NeuroImage, 47(2), 473–481. doi:http://10.1016/j.neuroimage.2009.04.056 19398013 10.1016/j.neuroimage.2009.04.056 PMC7201385

[hbm24194-bib-0031] Yücel, M. A. , Selb, J. , Aasted, C. M. , Lin, P.‐Y. , Borsook, D. , Becerra, L. , & Boas, D. A. (2016). Mayer waves reduce the accuracy of estimated hemodynamic response functions in functional near‐infrared spectroscopy. Biomedical Optics Express, 7(8), 3078–3088. doi:http://10.1364/BOE.7.003078 27570699 10.1364/BOE.7.003078 PMC4986815

[hbm24194-bib-0032] Zuo, X.‐N. , Di Martino, A. , Kelly, C. , Shehzad, Z. E. , Gee, D. G. , Klein, D. F. , … Milham, M. P. (2010). The oscillating brain: complex and reliable. NeuroImage, 49(2), 1432–1445. doi:http://10.1016/j.neuroimage.2009.09.037 19782143 10.1016/j.neuroimage.2009.09.037 PMC2856476

