# Supplementary figures and images for: Detailed view on slow sinusoidal, hemodynamic oscillations on the human brain cortex by Fourier transforming oxy/deoxy hyperspectral images
Source: Hum Brain Mapp. 2018 Apr 25;39(9):3558–73. doi: 10.1002/hbm.24194 (PMC6099526; doi:10.1002/hbm.24194)

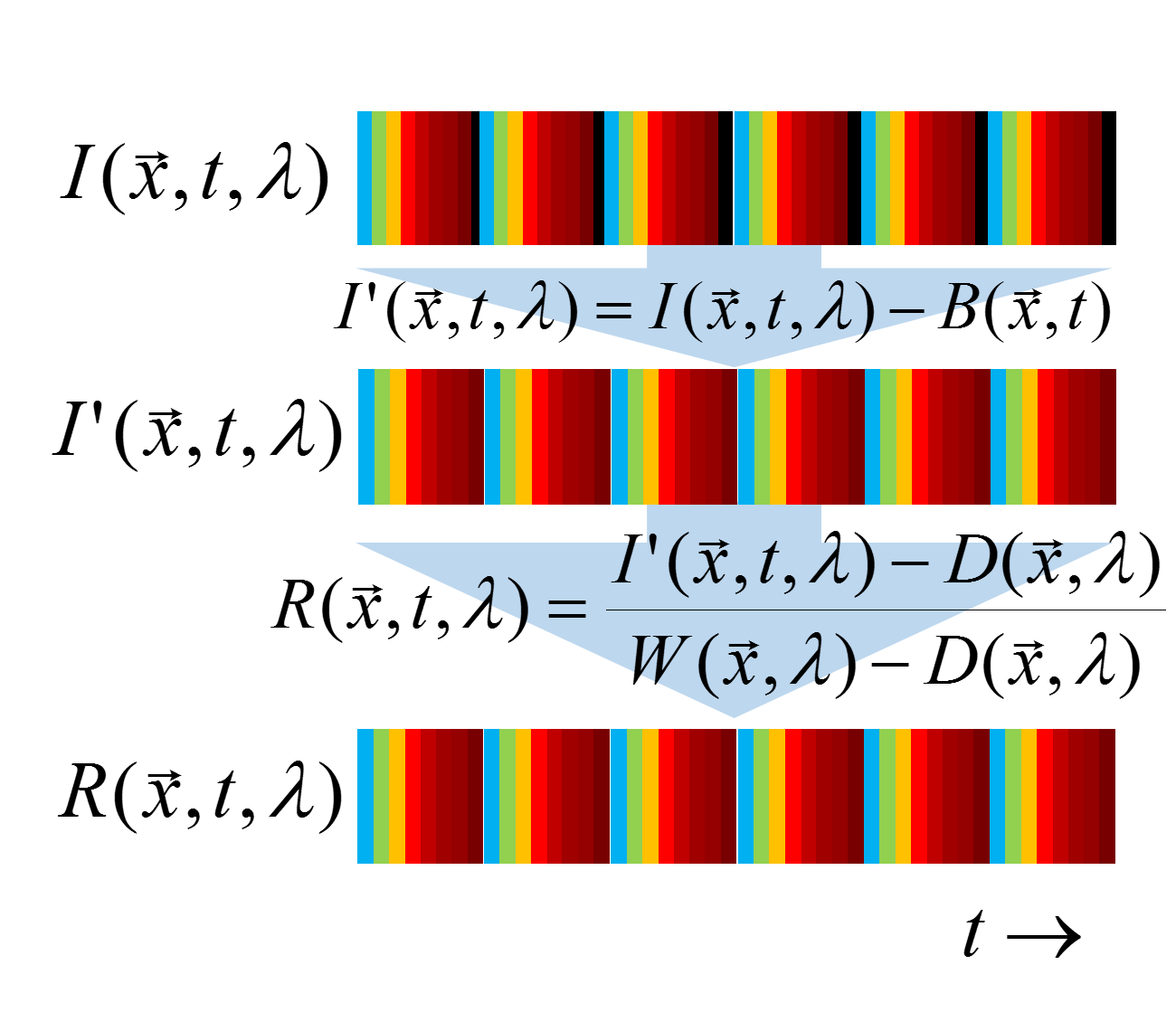

Supplement: Supplementary file 5 — Supporting Information [file HBM-39-3558-s005.tif]

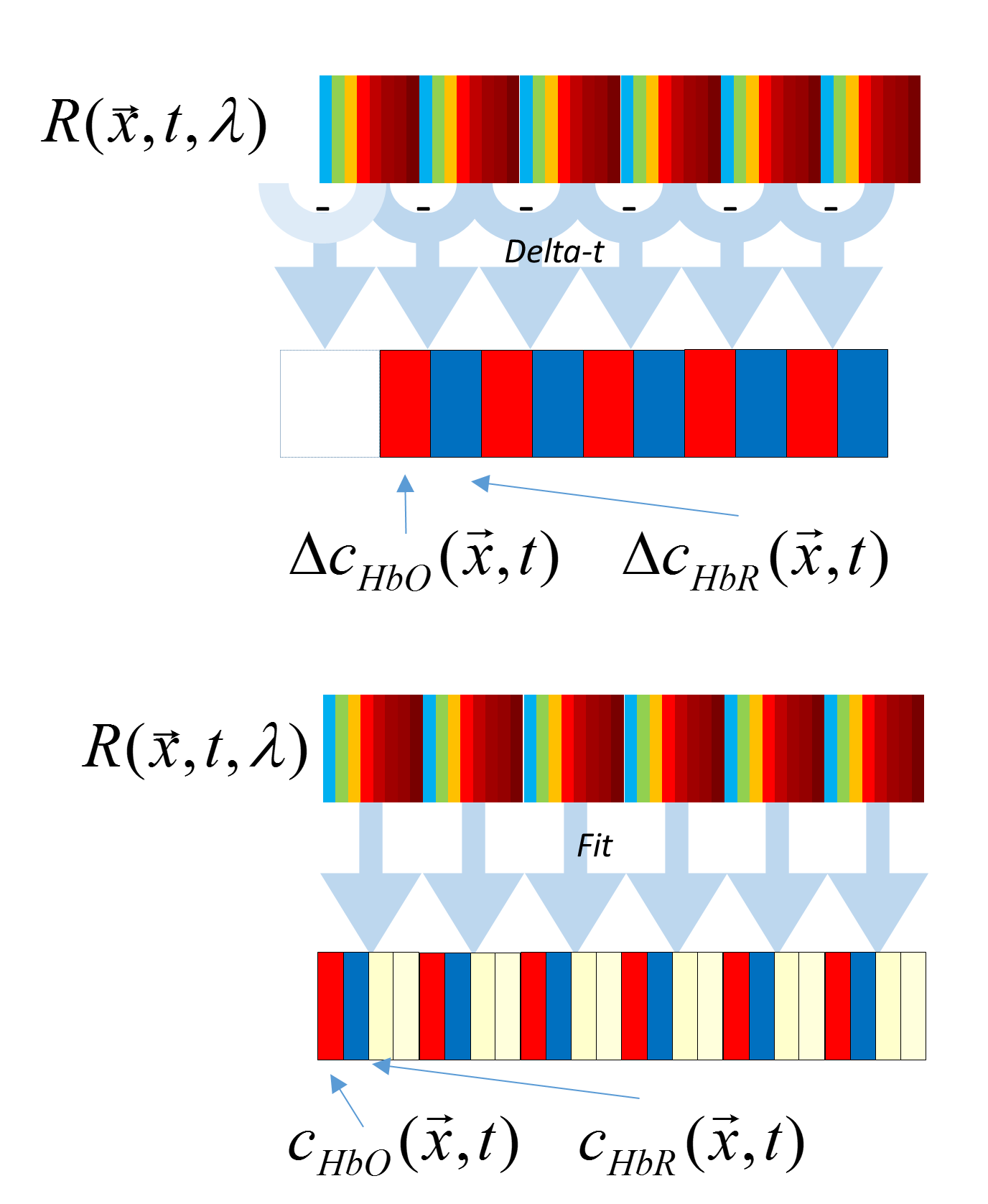

Supplement: Supplementary file 6 — Supporting Information [file HBM-39-3558-s006.tif]

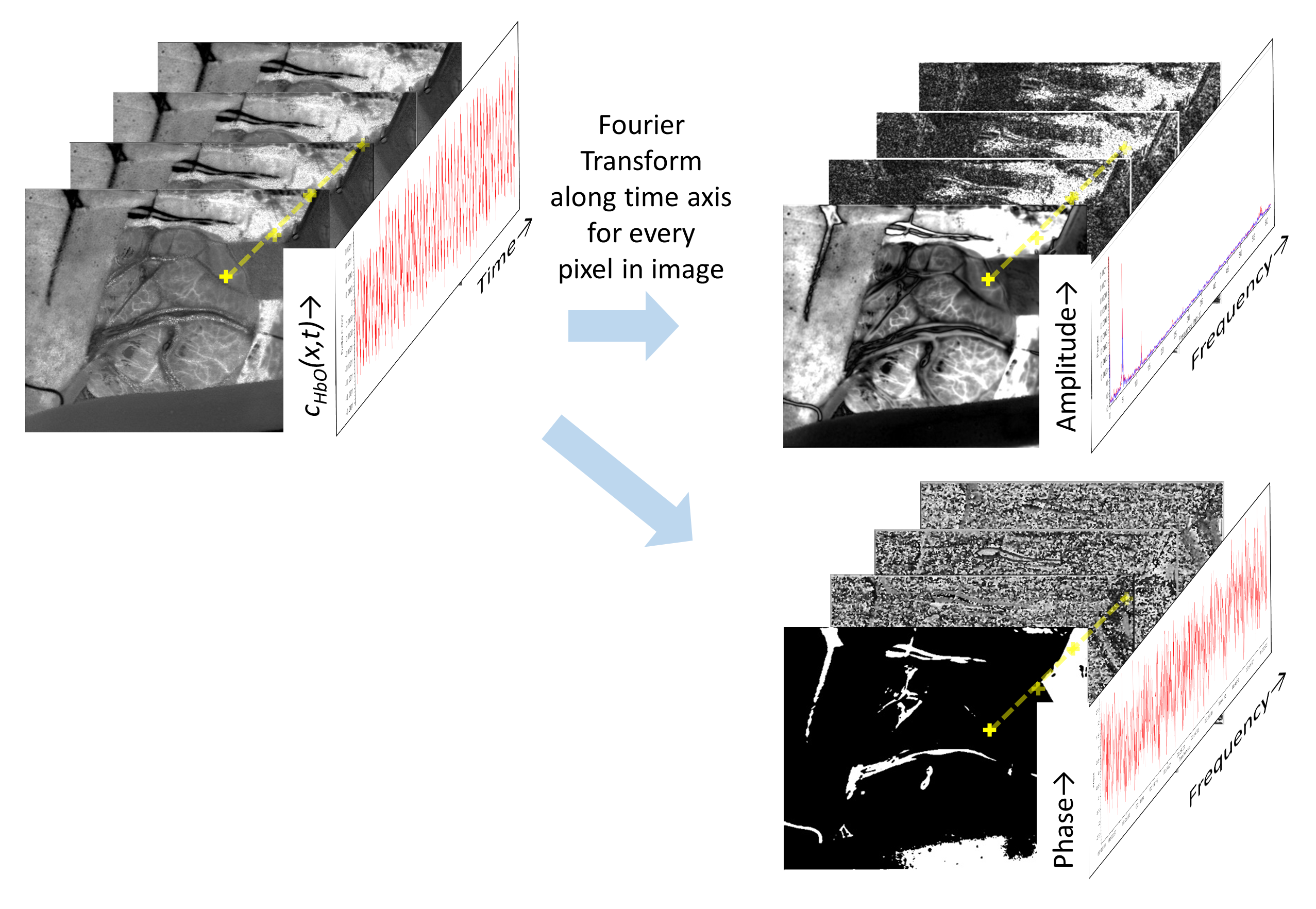

Supplement: Supplementary file 7 — Supporting Information [file HBM-39-3558-s007.tif]
