# Supplementary material for: Detailed view on slow sinusoidal, hemodynamic oscillations on the human brain cortex by Fourier transforming oxy/deoxy hyperspectral images
Source: Hum Brain Mapp. 2018 Apr 25;39(9):3558–73. doi: 10.1002/hbm.24194 (PMC6099526; doi:10.1002/hbm.24194)
Supplement: Supplementary file 8 — Supporting Information [file HBM-39-3558-s008.docx]

# Supplementary figures

Supplementary Figure 1 Calculation of reflection spectrum as pre-processing step for calculation of oxygenation concentrations. Explanation of symbols:

$I\left( \vec{x},t,\lambda\right):$ Intensity $I$ at pixel location $\vec{x}$, time $t$, and wavelength $\lambda$.

$B\left( \vec{x},t \right):$ Background intensity $B$ at pixel location $\vec{x}$, and time $t$.

$I'\left( \vec{x},t,\lambda\right):$ Background corrected intensity $I'$ at pixel location $\vec{x}$, time $t$, and wavelength $\lambda$.

$W\left( \vec{x},\lambda\right):$ White reference value $W$ at pixel location $\vec{x}$, and wavelength $\lambda$.

$D\left( \vec{x},\lambda\right):$ Dark reference value $D$ at pixel location $\vec{x}$, and wavelength $\lambda$.

$R\left( \vec{x},t,\lambda\right):$ Reflection value $R$ at pixel location $\vec{x}$, time $t$, and wavelength $\lambda$.

Supplementary Figure 2 Graphical representation of Fit and Delta-t methods to calculate absolute and relative concentration changes of oxy- and deoxyhemoblogin. Explanation of symbols:

$R\left( \vec{x},t,\lambda\right):$ Reflection value $R$ at pixel location $\vec{x}$, time $t$, and wavelength $\lambda$.

$c_{HbO}(\vec{x},t)$: Concentration of oxy-hemoglobin at pixel location $\vec{x}$, and time $t$.

$c_{HbR}(\vec{x},t)$: Concentration of deoxy-hemoglobin at pixel location $\vec{x}$, and time $t$.

$\Delta c_{HbO}(\vec{x},t)$: Change in concentration of oxy-hemoglobin at pixel location $\vec{x}$, and time $t$.

${\Delta c}_{HbR}(\vec{x},t)$: Change in concentration of deoxy-hemoglobin at pixel location $\vec{x}$, and time $t$.

Supplementary Figure 3 Calculation of amplitude and phase images of oxygenation concentration for every pixel in the image.

# Supplementary videos

Videos showing Fourier images along time axis

Patient 1 Most striking SSHO regions are visible around 0.049 Hz

Patient 2 One SSHO region visible at 0.093 Hz

Patient 3 Most striking SSHO regions at 0.082 and 0.090 Hz

Patient 4 Multiple SSHO regions between 0.065 and 0.112 Hz
